# The Identification of the Factors Related to Household Food Insecurity among Indigenous People (Orang Asli) in Peninsular Malaysia under Traditional Food Systems

**DOI:** 10.3390/nu10101455

**Published:** 2018-10-08

**Authors:** Leh Shii Law, Sulaiman Norhasmah, Wan Ying Gan, Adznam Siti Nur’Asyura, Mohd Taib Mohd Nasir

**Affiliations:** Department of Nutrition and Dietetics, Faculty of Medicine and Health Sciences, Universiti Putra Malaysia, Serdang 43400, Malaysia; lehshii@gmail.com (L.S.L.); wanying@upm.edu.my (W.Y.G.); asyura@upm.edu.my (A.S.N.A.); nasir.jpsk@gmail.com (M.T.M.N.)

**Keywords:** Orang Asli, food insecurity, Malaysia, challenges, in-depth interview

## Abstract

Over the course of 16 years, a high percentage of Orang Asli (OA) households in Malaysia has been found to be burdened with food insecurity. Therefore, a study was conducted to improve the understanding of the challenges faced by the OA in Peninsular Malaysia to achieve food security under traditional food systems. In this study, in-depth interview sessions, which were assisted by an interview protocol, were conducted with 61 OA women from nine villages that were selected purposefully across three states (Kelantan, Pahang, and Perak) in Peninsular Malaysia. Furthermore, thematic analysis was performed during data analysis. As a result, four themes were identified, namely (i) the failure in agriculture (sub-themes: threats from wild animals and insufficient land supply), (ii) ineffectiveness of traditional food-seeking activities (sub-themes: exhausting, tiring, dangerous, and time-consuming journey for food-seeking activities, depletion of natural commodities, reduced demands of natural commodities, and lack of equipment), (iii) weather (sub-themes: rainy and dry seasons), and (iv) water issues (subthemes: continuity of water supply and cleanliness of water). The identified modifiable factors of this issue should be incorporated into future schemes of food security intervention in order to efficiently manage the food shortage among the OA.

## 1. Introduction

Acknowledging food insecurity as a public health concern is a huge advancement in the chronological development of this issue. Several populations, namely children, the elderly, minorities, and low-income households have been known to possess a higher risk of facing food insecurity [1]. Furthermore, being the minority, Indigenous People are not excluded from this dilemma as the prevalence rate of food insecurity among this population has been reported to be high in some countries. Willows et al. [2] showed that 33% of Indigenous households in Canada were categorized as food-insecure. Within that total percentage, 14% of the cases were found to be severe. On the other hand, according to the Australian Institute of Health and Welfare [3], 24% of the Indigenous People in Australia, aged 15 or older, had faced food shortage in the previous 12 months from 2004 to 2005. Moreover, about 8% of the people affected by this issue claimed that they could not afford to buy food during a food shortage.

This issue was not widely explored by previous studies in Malaysia. The first research related to food security among the Orang Asli (OA) was conducted by Zalilah and Tham [4] who found that 82% of the OA households at Hulu Langat, Selangor were food-insecure. Meanwhile, Nurfahilin and Norhasmah [5] reported that 88% of OA households at Gombak, Selangor State had been food insecure until 2015. In addition, the most recent study on this matter revealed that 82.9% of the OA households (Mah Meri) at Carey Island and Tanjung Sepat (both are located in the Kuala Langat district) faced food insecurity; 29.3% household food insecurity, 23.4% individual food insecurity, and 30.2% experiencing child hunger [6]. Besides, Haemamalar et al. [7] and Chua et al. [8] found evidence related to food insecurity among the OA, which referred to the issue of the low dietary diversity (one of food insecurity indirect measures) among the OA in Pahang State, Malaysia.

Indigenous People, including the OA, are well-equipped with traditional knowledge inherited from generation to generation. This line of knowledge should have developed further with all the available natural resources offered by Mother Nature [9,10]. Therefore, the high prevalence rate of food insecurity among the Indigenous People is rather unexpected. This could be due to several challenges they encounter in practicing traditional food systems, which include the declining amount of plant and animal species, lack of transfer of cultural knowledge to youth, reduced time and energy invested for harvesting due to employment, decreased densities of species, and decreased land use. Moreover, the raising concern about environmental contamination and the increasing availability and acceptance towards new food are linked with the abandonment of traditional food systems in this community [11].

In addition, food security is closely linked with food systems. Food systems play a role in the food management process that consists of four stages including the production, processing and packaging, distribution and retail, and consumption of food. To put this simply, food systems regulate the overall process of food management [12]. Furthermore, the management of food loss and waste, which is another component under the food system, is a foundation of food security. Recently, significant food loss and waste has been reported during the harvesting, transportation, storage, packaging, wholesaling, retailing, and consumption of food [12]. Food loss and waste was estimated to be approximately one third of total food production globally (1.3 billion tons) [13]. The food loss and waste is not acceptable as millions of people are suffering food insecurity globally, yet tons and tons of food that were aimed to feed the targeted groups were wasted due to mismanagement [12]. This also implies that presence of food availability alone does not guarantee food security. The food should be made (physically, economically, and socially) accessible to the public [14]. Therefore, the primary focus of this study is the traditional food system. The traditional food system for Indigenous People is described as “being composed of items from the local, natural environment that are culturally acceptable” [11] (p. 417). An in-depth exploration of the traditional food system is necessary, provided that most of the Indigenous People worldwide depend on the food systems to obtain food and generate income [9]. 

Based on the data obtained from the Department of Orang Asli Development (JAKOA), the population of OA in Peninsular Malaysia was 178,197 in 2013, and this number was divided into three main ethnic groups, which were Senoi, Malay-Proto, and Negrito [15]. Orang Asli were expected to flourish under the traditional food systems attributed to their rich knowledge of their surrounding environment (forests/lakes/rivers) and food-seeking activities (farming/hunting/gathering). However, contradictive findings from other studies showed a high prevalence of food insecurity and low dietary diversity among the OA in Peninsular Malaysia. This information proves that the OA are facing hardships to obtain sufficient food through traditional food-seeking methods, namely farming, hunting, fishing, and gathering. Therefore, drastic measures are needed to curb food insecurity issues among the OA. Before any recommendations and resolutions can be planned or proposed, a thorough understanding of this issue is important. For this reason, this study was conducted to identify the factors connected with food insecurity among the OA. This study aims to find out the factors for the absence of traditional food practices as a safeguard for OA to obtain enough food.

## 2. Materials and Methods

This is a qualitative research study using a case study design. According to Yin [16], a case study is an empirical enquiry into answering the “how” and “why” research questions. The focus of the case study is the contemporary phenomenon in real life contexts where investigators have little to no control over real-life events. The particular case concerned in this study is the occurrence of household food insecurity, while the unit of analysis is the ‘hardcore poor’ OA households.

### 2.1. Study Location

**Figure 1:**
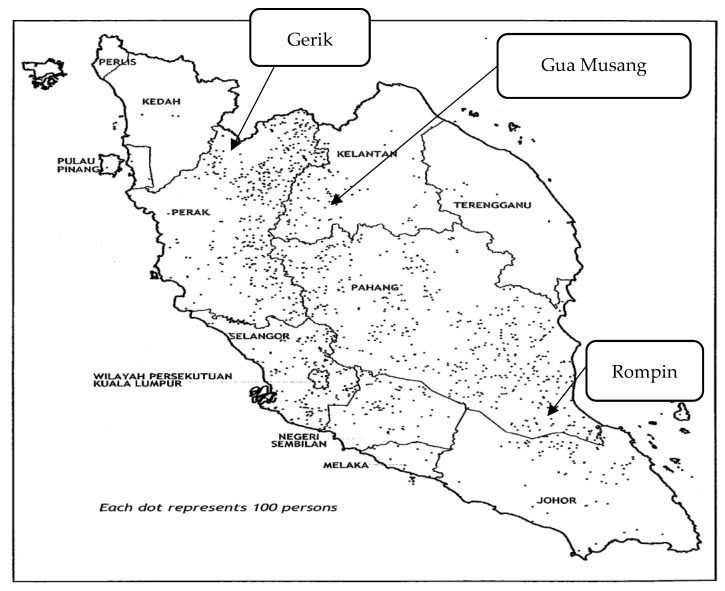
Distribution of *Orang Asli* in Peninsular Malaysia, 2000.

The locations for this study were selected purposefully based on the number of the food assistant recipients under JAKOA. The list of food recipient names from each administrative region was obtained from the JAKOA state headquarters or the JAKOA administrative offices. The administrative regions were then divided into three groups, namely Senoi dominant, Proto-Malay dominant, and Negrito dominant. Then, one district was selected under each ethnic cluster. The villages from each district were also purposefully selected using the priority given to the particular villages with a high number of food assistant recipients. However, the provision of this priority was subjected to the advice given by the JAKOA staff as they were more familiar with the conditions in the villages. The selected study locations were at Gua Musang District (Kelantan State), Rompin District (Pahang State), and Gerik District (Perak State). The study locations are shown in the Figure 1 [17]. 

Gerik is a *mukim* (administrative division) of Hulu Perak, a district at the north of Perak State. Based on the information provided by JAKOA, a majority of the OA here are Negrito. Meanwhile, Gua Musang is a district at the southern part of Kelantan State, with Senoi as the biggest community there. Lastly, Rompin is a district located at the southeast corner of Pahang State, and Proto-Malay appears to be dominant in this region. 

### 2.2. Informants

The informants consisted of 61 OA women. The inclusion criteria was that they were of child-bearing age (20–49 years old), brought along at least a single child during data collection, and lived in ‘hardcore poor’ households that received food assistance from JAKOA. According to JAKOA, the classification of the households as ‘hardcore poor’ was based on the guidelines provided by the Economic Planning Unit [18] which stated that the households which receive an income below Malaysian Ringgit (MYR) 520 per month (United States Dollar (USD) 127.45 (10 August 2018)) are classified as ‘hardcore poor’. The sample size of the informants was based on the concept of saturation. A stage of saturation was achieved when no new information emerged as the interview progressed [19].

### 2.3. Ethical Clearance and Permissions

Prior to data collection, ethical clearance was obtained from the Ethics Committee for Research Involving Human Subjects (JKEUPM) of Universiti Putra Malaysia (Serdang, Malaysia) [Reference no.: FPSK(EXP15)P004]. The permission to carry out this study at the OA villages was obtained from JAKOA. At the same time, all the informants needed to sign a written consent form to prove their willingness to voluntarily participate in this study. However, they were allowed to withdraw from the study without any penalty, and all the information provided by the informants were kept confidential.

### 2.4. Data Collection

This process was conducted through several sessions of semi-structured in-depth interviews with the help from an interview protocol. The interview protocol referred to a list of questions and issues of interest that were covered during the in-depth interview. The interviews were audio-recorded with permissions from the informants. The Malay language, a national language among Malaysians regardless of their ethnic background, was used during this session. However, for informants who were not able to express themselves in this language, the services of translators were used to smooth the interview sessions, and they consisted of the local OA who were able to speak both the native OA language and the Malay language. Another instrument used in this study was a sociodemographic questionnaire that was used to assess the sociodemographic characteristics of the informants.

Each of the informants was required to go through two sessions of the in-depth interview. The primary aim of these sessions was to establish a sense of rapport between the researcher and the informants [20]. To reemphasize, this study particularly looked into the contributing factors of household food insecurity under traditional food systems. The questions which were directed to the informants were:

- Are you facing any problems in obtaining sufficient food?
- (Based on Question 1) If yes, can you elaborate the problem(s) you are facing by providing a suitable example(s)?
- (Based on Question 2) How frequent is/are the problem(s)?

### 2.5. Quality Control

In order to ensure the authenticity of the information extracted from the informants, the plan to involve the heads of the villages or renowned individuals during data collection was implemented. Based on the observation prior to the test, the presence of the Tok Batin (heads of the villages) or renowned individuals helped to get rid of suspicion of information. Furthermore, translation services also helped smooth the process of the interviews. Both approaches reduced the potential biases among the informants, be it intentional or unintentional, such as providing inaccurate information due to misinterpretation of the questions. Triangulation was another valid method used to secure the information gathered from the interviews. Data triangulation (the involvement of all three main ethnic groups of OA) and environmental triangulation (the involvement of three study locations) were incorporated into the sampling methods [21]. The information provided by all the three ethnic groups were considered to have the highest authenticity.

### 2.6. Data Analysis

The findings of the numeric sociodemographic characteristics were presented with medians, while the category sociodemographic variables were presented in counts and proportions. The descriptive data of the informants were analyzed using IBM SPSS Statistics version 22.0 (IBM Corp., Armonk, NY, USA). On the other hand, the extraction of the information from the interview was done through thematic analysis. This action was based on the techniques guide from Braun and Clark [22]. First, the verbal data from the interviews were transcribed verbatim. The transcription process helped researchers familiarize themselves with the data [23]. Then, the transcripts were read repeatedly in order to determine the breadth of the data. Normally, the repeated reading is followed by other processes such as note-taking and marking the ideas for the coding process that follows after. Through the familiarization of data conducted in the previous step, the coding process starts. In this study, the coding process was carried out manually using highlighters or colored pens. The codes identified from the data familiarization were matched up with the data extracted from individual transcripts. These transcripts were found to demonstrate the characteristics of the code. Each code that was identified, including the relevant data extracts, was kept in a separate computer file. Following that, the identified codes were categorized into potential themes/sub-themes, while the relevant coded data extracts were categorized into the identified themes/sub-themes.

## 3. Results

### 3.1. Characteristics of the Informants

A total of 61 informants from three districts (Gua Musang, Rompin, and Gerik), with a median age of 32 (range: 20–49) years, were recruited. Most of the informants were from big households where four or more children resided. Furthermore, a high percentage of the informants either had a low formal education or did not pursue a formal education at all. As for their socioeconomic characteristics, most of the informants were housewives, while most of their spouses were skilled agricultural, forestry, and fishery workers. Subsequently, the selected households were then categorized as ‘hardcore poor’ except for a single household, which was labeled as ‘poor’. Further details regarding the informants’ demographic and socioeconomic characteristics are presented in Table 1.

### 3.2. Factors Related to Household Food Insecurity

Meals of OA comprised mixtures of market and traditional food due to the variances in their food-seeking behaviors. Market food was purchased from nearby food outlets, including rice, flour, biscuits, tea, cereal, and green leafy vegetables. Traditional food was usually obtained through food-seeking activities and farming that included gathering edible fruits and vegetables (stinky bean, fern shoot, and natural herbs), fishing (local fish and prawn), hunting (wild boar, squirrel, and lizards), farming (tuber roots, rambutan, and durian), and raising poultry (chicken and duck). In brief, traditional food was obtained from their surrounding environment without involving money.

The thematic analysis revealed four potential themes which contributed to household food insecurity among the OA under traditional food systems. The aforementioned themes were the failure in agriculture, the ineffectiveness of traditional food searching methods, water issues, and weather.

#### 3.2.1. The Failure in Agriculture

In the case of the failure in agriculture, the informants expressed their concerns of the agricultural products cultivated from their farms being under the threat from wild animals, such as wild boars, monkeys, and rats if not guarded properly. The wild animals that invaded the farms, not only stole the agricultural products, but also destroyed the plants. It was reported that this case was faced by the Senoi and Proto-Malay groups only. 

Informant 15:

“The products were eaten by the foxes and the birds. Yes. Today, when I went there (farm), everything seemed fine. However, tomorrow or the day after, when I went there, the wild boars might have eaten the products. I guard my farm myself. I do the patrol around the farm. I find the rattans, cut the bamboos, to build a guard post. I guard it myself. There are animals out there which eat our products. Sometimes, when we plant tuber roots, the wild boars eat it. As for pumpkins, the insects from the forest eat them. The insects eat… the grasshoppers eat the shoots of the tuber plants until nothing is left behind.”

Informant 42:

“It (wild boar) eats the tapioca. It eats the sweet potatoes. It eats the banana tree. If we plant the pineapple, it also eats the pineapple. Nowadays, there are many wild boars.

In addition, the informants were concerned with the lack of arable land for them to carry out self-sustainable agricultural activities. Orang Asli did not possess the arable lands nearby and the lands were taken to run domestic agricultural activities and mining. The Senoi and Proto-Malay were the only groups who were reported to face this issue:

Informant 3:

“For example, I really do not own any piece of land. It is not belonged to me. Whatever farms you can see here are all belonged to the other people.

Informant 50:

“Not enough land. Only have a small piece of land. Do not have new land because all have been cleared up to become the (domestic) farms.”

#### 3.2.2. The Ineffectiveness of Traditional Food-Seeking Methods

Besides the failure in agriculture, the ineffectiveness of traditional food seeking methods was found to be another theme which explained the failure of the OA to achieve household food security. This is because this method only depends on wild plants and animals from their surrounding environment. Furthermore, food gathering and hunting were described as time-consuming, dangerous, uncertain, and exhausting. Despite the hard work, traditional food-seeking activities did not always guarantee enough food. These factors were reported by all three ethnic groups involved during the interview. 

Informant 7:

“(Leaves) at six o’clock in the morning, comes back at seven o’clock, or eight o’clock at night. If we travel through the forest at seven in the morning, will come back at three or four in the afternoon.”

Informant 21:

“When my husband travels to the forest, there are a lot of challenges awaiting him, such as the presence of elephants, tigers, snakes, and centipedes.”

Informant 13:

“Searching for food is not easy. Sometimes, the food that we wish to find is available, but sometimes it is not. Especially when we go fishing. Sometimes the fish bites, and sometimes we have to go back home with empty hands.”

Informant 52:

“The road… the road is covered with mud. We need to go through the bushes too. Sometimes, the walking path is covered by stones. Climbing up and down, up and down the hills.”

Due to deforestation, the condition of the food insecurity among informants worsened due to the depletion of natural commodities in the forests. Forests provide habitat for various flora and fauna. Many species that OA depended on for food cannot survive due to loss of home. This factor was reported by all three ethnic groups (Senoi, Proto-Malay, Negrito). 

Informant 2:

“It (the fern shoot) is not available here, very hard to find. Want to eat (wild) vegetables, but it is not available. (The place with fern shoots is) only reachable by a motorcycle as it is far away. Do not have (wild vegetables here), if you find at surrounding here, (you) only manage to get a few, three or four sticks. Not enough to eat.”

Informant 16:

“Difficult. It was quite easy in the old days, but now, it is hard. Due to some people who conduct the mining activities, and because of that, the natural products of the forest are no longer available. At the side (of the forest) where people conduct the tin ore mining activities, the forest will be demolished.”

Furthermore, the demand for natural commodities, such as herbs, was no longer popular compared to the demand for these resources from decades ago. This led to the drop in the price of the natural commodities. This factor was reported by all three ethnic groups (Senoi, Proto-Malay, Negrito). 

Informant 32:

“It is not sold at high price, not very high and sometimes it is sold in low price. Take the herbs with medical value from the forest as an example, we get three Ringgit fifty cents for a kilogram. If we manage to get a few kilograms, we get a few dozen Ringgit.”

Informant 57:

“Only my husband knows about the price of the natural commodities. The price was only known before though. Nowadays, no one has been requesting for natural herbs. For example, people requested Tongkat Ali (a traditional medicine) only during the old days. It is no longer requested now.”

The informants were also reported to abandon hunting activities due to the lack of the equipment needed. Weapons were needed to hunt big-sized animals (e.g., wild boar and deer). This problem was found to occur among all ethnic groups, namely Senoi, Proto-Malay, and Negrito. 

Informant 42:

“I do not have an equipment for fishing. How can I go fishing? (Besides that, it is) hard to get wild animals. No gun is available for hunting. (We) do not have an equipment (for the activities).”

Informant 50:

“It is because we do not have the equipment for fishing. We do not have the gun for hunting.”

#### 3.2.3. Water Issues

It was found that the *Orang Asli* in Malaysia were still burdened with the problems of obtaining sufficient clean water supply which explained the failure of the OA to achieve household food security. Water issues were labeled as the third theme under factors associated with household food insecurity among OA. To be specific, the water supply was reported to be inconsistent due to the blockage of the pipe system, especially after the rain. Besides, agricultural and logging activities became a concern as these activities contaminated the rivers or lakes which were known to be the primary water sources for the OA. The river became muddy especially after raining due to deforestation. Meanwhile, the OA were worried about the possible leakage of pesticides used for domestic agriculture activities into the rivers. This problem was reported by all ethnic groups in the present study.

Informant 56:

“For me, if I want to declare the water supply that I got is clean is not true. (The water) may be considered moderately clean. The problem is due to land clearing and logging, water is affected and is no longer clean. When it rains, water looks muddy.”

Informant 59:

“Water supply? We are not satisfied with it. Because when some people performing logging activities, the water will be exposed to pollution. When it rains… during the dry seasons, the water looks a bit clear. However, when it rains, it looks a bit muddy. Therefore, it is hard for us to get obtain a clean water supply. Water supplies cannot be continued as sometimes the pipe is blocked during the rain and we need to go repair it. Therefore, the actual problem is the blocked pipe, not the water.”

#### 3.2.4. Weather

This issue fell under the fourth theme which explained the failure of the OA to achieve household food security. To be specific, Malaysia experiences two monsoon seasons, namely the southwest monsoon (June to September) and northeast monsoon (November to March) as well as two inter-monsoon seasons (October and April to May). The southwest monsoon brings rain to the east coast while the northeast monsoon increases rainfall to the west coast of Peninsular Malaysia [24]. The extreme rainy season was found to interrupt economic activities, such as rubber tapping, mining, and searching for natural commodities in the forests. Furthermore, heavy rain also led to the occurrence of floods which destroyed the farms cultivated by the OA. This problem was found to occur among all ethnic groups in the present study.

Informant 46:

“Due to the raining season, we become jobless. It is hard to find a job. Some of the available jobs are related to farm works, such as rubber tapping. These jobs are usually pursued through contracts and recruits are unneeded due to rain. Workers are recruited only during dry seasons. This is why sometimes earning money is not an easy task for us.”

Informant 52:

“A bit tough when the rainy season arrives. Cannot earn money. For example, we want to search vegetables or want to harvest the banana for sale are also restricted, people (traders) do not want the products. In addition, people (traders) do not travel here to pick up the products.”

On the other hand, the extreme dry season reduced the chance of successful harvesting as crops or vegetables might die before the plants got to be full grown. This factor was also reported by all ethnic groups in the present study.

Informant 4:

“Dry season. The leaves wither instead of flourishing. The land is dry, but many still carry out agricultural works. Harvesting can be done, but the plants will not fully grow.”

Informant 45:

“Water, sometimes do not have water. The water flow is slow. The tuber roots are also dying. Because of the heat.”

## 4. Discussion

The findings provided evidence regarding the presence of household food insecurity among the OA, especially among those with the low socioeconomic background. Overall, the factors of household food insecurity under traditional food systems included the failure in agriculture, the ineffectiveness of traditional food seeking methods, water issues, and weather. Presence of challenges in conducting traditional food-seeking activities implies that food sovereignty of the OA was violated due to deforestation and pollution of the water sources. Orang Asli were found to have no control over their territories where they obtained their food (forests, rivers, lakes, and arable lands nearby their villages) from destruction as a consequence of the economic activities that were conducted by third parties [25,26]. This finding is important for a better understanding of the hardships faced by the OA so that counteracting policies can be implemented for an improved standard of living. 

The first factor of household food insecurity among the OA was the failure in agriculture. Most of the OA cultivated their own farms either at the nearby forests or beside their houses. However, their farms were always under the threat of wild animals, such as wild boars. A similar finding was reported by Zakari et al. [27] as the occurrence of diseases and presence of insects in the farms were found to be related with household food insecurity among the farmers at Kollo district, Southern Niger. Besides, some informants reported that insufficient agricultural lands disrupted the OA’s self-sustainable agricultural activities which should have been done to support the needs of their household members. As a result, the vulnerability to food insecurity of the households that owned no or small agricultural lands increased. This happened as the nearby lands in the villages were taken over for the development of mining sites, logging sites, or cash crop plantations, such as palm oil trees and rubber trees [28]. Similarly, insufficient agricultural land puts a limit on food availability for household members’ daily consumption, and it dwindles the economic income that can be generated from the cultivation of household farms [29]. The importance of land in preventing household food insecurity by increasing the household income was reported by Kirimi et al. [30] in Kenya, who pointed out that the ownership of lands for households was found to reduce the risk of poverty (low household income to fulfil the needs of the basic food and non-food necessities) and help the households overcome food insecurity. 

Naturally, OA are well-known for their capability of finding their own food from their surroundings, either from the forests, rivers, or lakes. One of the factors which was believed to have led to the obstacles faced by the OA in finding sufficient food is the depletion of food resources. This is the result of deforestation due to logging activities, either conducted legally or illegally. Furthermore, Malaysia had reported an increasing forest loss from 2000 to 2012 [31]. Not only that, insufficient proper equipment restricts the OA’s from carrying out hunting activities for food. Similar challenges were also found to occur to other groups of Indigenous People worldwide. For example, a study conducted on Vuntut Gwitchin First Nation from the Old Crow and Tlingit households in Teslin, Canada found that insufficient time and limited availability of traditional food were the factors that disrupted the continuation of hunting activities. Other factors that disrupted the continuation of hunting activities included the unaffordable price of harvesting tools, cultural loss, and the concern of contamination [32]. In addition, the challenge faced by the Inuit communities at Pangnirtung, Nunavut, Canada was that their activity of food-searching around their surroundings was disrupted due to climate change, environmental degradation, market economy, globalization, acculturation, and nutrition transition [33].

There were two main issues associated with water supply, namely the disruption of the water supply and the concern for the cleanliness of the water. The disruption of the water supply was mainly due to the lack of maintenance and blockage of personal water pipes by sand or dry leaves after the rain, which prevented water from flowing to the reservoir. The reservoir is where all the villagers obtain water. In the case of the cleanliness of water, the informants voiced their concern about the pollution of the river near their area due to logging and agricultural activities [34]. A shift in the pattern of diseases among OA such as hepatitis A and B, tuberculosis, and dermatological and gastroenterological problems was related to the use of polluted water [35]. Besides, many other diseases were associated with this pollution issue, which included skin diseases, diarrhea, dysentery, respiratory illnesses, anemia, and complications in childbirth [36]. Therefore, water issues among the OA should be taken seriously in order to prevent these illnesses from spreading.

In the recent years, Malaysia faced several episodes of extreme weather events, such as floods, droughts, and heat waves [37]. Provided that the OA’s quality of life is dependent on their surrounding environment, the weather is believed to be another factor which may determine the status of their household food security. Moreover, the interaction between extreme climate and the status of household food security was shown in Bahiigwa’s [38] study, which demonstrated a fluctuating pattern in the prevalence rate of food insecurity in 1000 households from four geographical regions in Uganda (western, central, eastern, and northern). This was due to the lack of rainfall, pests and diseases, and excessive rain. Likewise, Niles and Salermo [39] revealed that households from 15 countries in Latin America, Africa, and South Asia that faced climate shock had a higher likelihood to experience household food insecurity. Meanwhile, the Food and Agriculture Organization [40] showed that climate had the potential to influence the quantities and types of food produced and the amount of the production-related income. At the same time, the extreme weather might have adverse impacts as it could destroy the transportation and distribution facilities, and it could increase the risk of food insecurity among the vulnerable groups within the community. Besides, unfavorable rainfall patterns and temperature were reported to lead to the decline in the availability of wild food that is needed by many families in the midst of food shortage. 

There are several limitations to this study. The villages selected for this study were located where researchers could easily travel to by normal car. This indicated that the village location was connected to the nearby town by several roads. For this reason, the informants did not only depend on traditional food systems for food-seeking and gaining access to food markets. Therefore, one of the recommendations is that the OA from both remote and suburban villages should be involved in similar future studies. The other limitation of this research could be seen from the participants who were limited to female informants. In contrast, the involvement of male informants may improve the information obtained in a study through different question sets being used for male and female informants [20]. To be specific, the questions for male informants could focus on the socioeconomic activities of the households, while the questions for female informants could focus on the dietary patterns of the household members. Another limitation of this study was the use of a translation service, which might affect the validity of information obtained from the informants. In this case, translators might unintentionally provide unnecessary hints to the informants at some point in the interview. In order to prevent such biases from occurring, translators should be properly trained prior to data collection, and they need to always be reminded to not provide additional explanation to the informants.

## 5. Conclusions

In conclusion, the OA generally rely on the knowledge they inherit from their ancestors regarding nature for their survival. The traditional food systems are essential for the OA to obtain food for the daily consumption of the household members. However, due to the negative effects in the midst of urbanization, the availability of food in the natural environment to support their livelihood has been reduced. As a result, the OA have shifted to modern food systems, where food is purchased from the markets to fulfill their needs. According to the recent trend, the deterioration of the traditional food systems is due to five factors, which include the failure in agriculture, the ineffectiveness of traditional food-seeking methods, water issues, and weather. These are the important findings which have distinguished the contexts of the OA’s household food insecurity from the contexts of the general population. Moreover, the findings of this study have proven that household food insecurity among the OA is an existing phenomenon, and a clear picture of the issue has been provided to demonstrate the unfortunate situation faced by this community. With this, it is recommended that local authorities and non-governmental organizations take this issue seriously due to the hazardous impacts it can bring to the health of its victims. Through a proper understanding of the household food insecurity among the OA, drastic measures could be taken by the responsible authorities in order to relieve the OA from this problem.

## Figures and Tables

**Table 1:** Sociodemographic characteristics of the informants (*n* = 61).

Variable	Count (*n*)	Percentage (%)	Median (Range)
Age (years)			32 (20–49)
20–29	26	42.6	
30–39	22	36.1	
40–49	13	21.3	
Ethnicity			
Senoi (Temiar)	20	32.8	
Proto-Malay (Temuan)	20	32.8	
Negrito (Jahai)	21	34.4	
Religion			
Islam	27	44.3	
Christianity	8	13.1	
Animism	26	42.6	
Marital Status			
Married	57	93.4	
Widowed	4	6.6	
Number of Children			3 (1–13)
≤2	24	39.3	
3–5	19	31.1	
6–8	13	21.3	
≥9	5	8.2	
Education Level of Informants			1.5 (0–11.0)
No formal education	25	41.0	
Primary school	21	34.4	
Secondary school	15	24.6	
Education Level of Spouses			2.5 (0–11.0)
No formal education	24	39.3	
Primary school	26	42.6	
Secondary school	10	16.4	
Do not know	1	1.6	
Occupation of Respondents			
Skilled agricultural, forestry, and fishery worker	12	19.7	
Plant and machine-operators and assembler	1	1.6	
Housewife	48	78.7	
Occupation of Spouses			
Skilled agricultural, forestry, and fishery worker	52	85.2	
Craft and related trade worker	1	1.6	
Plant and machine-operators and assembler	3	4.9	
Elementary occupation	1	1.6	
Divorced	1	1.6	
Passed away	3	4.9	
Monthly household income (MYR *)			200.0 (50.0–750.0 ^†^)
Income per capita (MYR *)			41.7 (8.3–250.0)
Expenses of food and beverage (MYR *)			100.0 (20.0–250.0)

* USD = United States Dollar, MYR = Malaysian Ringgit; USD 1 = MYR 4.08 (10 August 2018). ^†^ A household was grouped under “poor” (monthly household income between MYR 520 and 830) due to job opportunity weeks before interview. The household was in the name list of food assistant recipient in 2014. Therefore, the household was eligible for this study.
